# Is Your System Calibrated? MRI Gradient System Calibration for Pre-Clinical, High-Resolution Imaging

**DOI:** 10.1371/journal.pone.0096568

**Published:** 2014-05-07

**Authors:** James O’Callaghan, Jack Wells, Simon Richardson, Holly Holmes, Yichao Yu, Simon Walker-Samuel, Bernard Siow, Mark F. Lythgoe

**Affiliations:** 1 UCL Centre for Advanced Biomedical Imaging, Division of Medicine, London, United Kingdom; 2 UCL Centre for Medical Image Computing, London, United Kingdom; Maastricht University Faculty of Health, Medicine, and Life Sciences, Netherlands

## Abstract

High-field, pre-clinical MRI systems are widely used to characterise tissue structure and volume in small animals, using high resolution imaging. Both applications rely heavily on the consistent, accurate calibration of imaging gradients, yet such calibrations are typically only performed during maintenance sessions by equipment manufacturers, and potentially with acceptance limits that are inadequate for phenotyping. To overcome this difficulty, we present a protocol for gradient calibration quality assurance testing, based on a 3D-printed, open source, structural phantom that can be customised to the dimensions of individual scanners and RF coils. In trials on a 9.4 T system, the gradient scaling errors were reduced by an order of magnitude, and displacements of greater than 100 µm, caused by gradient non-linearity, were corrected using a post-processing technique. The step-by-step protocol can be integrated into routine pre-clinical MRI quality assurance to measure and correct for these errors. We suggest that this type of quality assurance is essential for robust pre-clinical MRI experiments that rely on accurate imaging gradients, including small animal phenotyping and diffusion MR.

## Introduction

Pre-clinical, high-field MRI systems are now widely used to provide high-resolution images of animal models of human disease. Such phenotyping studies have enhanced our understanding of numerous disease processes and, when used in combination with advanced computational methods, have been used to detect subtle differences in tissue structure, particularly in the brain [1]. To perform accurate, longitudinal, phenotyping studies, both *in vivo* and *ex vivo*, requires careful calibration of the MRI scanner, in particular of imaging gradients, so that microscopic changes in tissue structure can be robustly measured.

Imaging gradients are generally calibrated via linear scaling factors in each principal imaging axis, and any error in their values results in a global compression or expansion of acquired images. Furthermore, imaging gradients are assumed to be spatially linear, and complex image distortions can be introduced by gradient non-linearity, which rapidly manifest with increasing distance from the magnet isocentre. Gradient calibration is typically performed annually during routine maintenance by the scanner manufacturers. Scaling factors and linearity are assessed using a structural phantom. Distances between structures are measured on a single slice of an MRI image of the phantom and compared to those of the original CAD design. This approach is highly vulnerable to error due to operator variability, the small number of measurements taken and the large slice thickness (1 mm) used. Neither drift of applied gradients over time due to hardware instability between maintenance visits nor deformations in the phantom structure over time are accounted for. Additionally, acceptance limits specified (≤5% linearity over a 40 mm diameter of spherical imaging volume (DSV)) are potentially unsuited to the degree of accuracy required for phenotyping studies.

Whilst several strategies for calibrating imaging gradients have been proposed for clinical scanners [2], [3], [4], [5], these may not translate to pre-clinical scanners which differ from clinical scanners in several fundamental ways. For example, the maximum imaging gradient strengths are typically an order of magnitude greater than those found on a clinical system, scanners have a much smaller bore size, and field strengths are typically between 7 and 11.7 T, compared with 1.5 or 3 T for clinical systems. Due to the challenging design considerations, large distortions can be observed in pre-clinical gradient sets. In these scenarios, control point or spot matching algorithms employed in clinical protocols to unwarp distorted images have been shown to fail and require operator intervention [6]. Furthermore, the level of accuracy required for phenotyping studies (of the order of 10 s of microns) outweighs that typically required for clinical *in vivo* studies.

We therefore aimed to develop a gradient calibration and quality assurance protocol for pre-clinical MRI scanners, which can be customised to individual systems and RF coils, and that aims to reduce geometric distortions and ensure stability over time in both *in vivo* and *ex vivo* longitudinal phenotyping studies. Moreover, studies using imaging techniques that also rely on accurate gradients (such as diffusion MRI) would also benefit.

The protocol is based on a structural phantom that was constructed and designed in-house. We used 3D printing to construct the phantom which is a fast, straightforward and cost-effective method to build bespoke components [7], particularly of the size required for pre-clinical systems. Materials were chosen to be susceptibility matched, robust, and usable in successive studies. CAD plans for our phantom are open-source, and have been published online: https://www.ucl.ac.uk/cabi/publications/open_source.

The calibration protocol consists of two stages: i) a system calibration method for absolute scaling of imaging gradients; ii) a post-processing correction of gradient non-linearity, achieved through non-rigid image registration. All post-processing software is feely available to download (*NifTK*, http://cmic.cs.ucl.ac.uk/home/software/), making this simple protocol easy to adopt as part of a pre-clinical quality assurance (QA) procedure.

In this study, we introduce the protocol and evaluate to what extent it can improve the accuracy of imaging gradients, compared with the values derived from the standard calibration procedure performed by the scanner manufacturer. We also monitor the variation in the calibration parameters across a six-month period, alongside a serial assessment of the structural stability of the phantom.

Here follows, a brief, step-by-step description of the calibration protocol.

### Gradient Calibration Protocol Description

**Figure 1:**
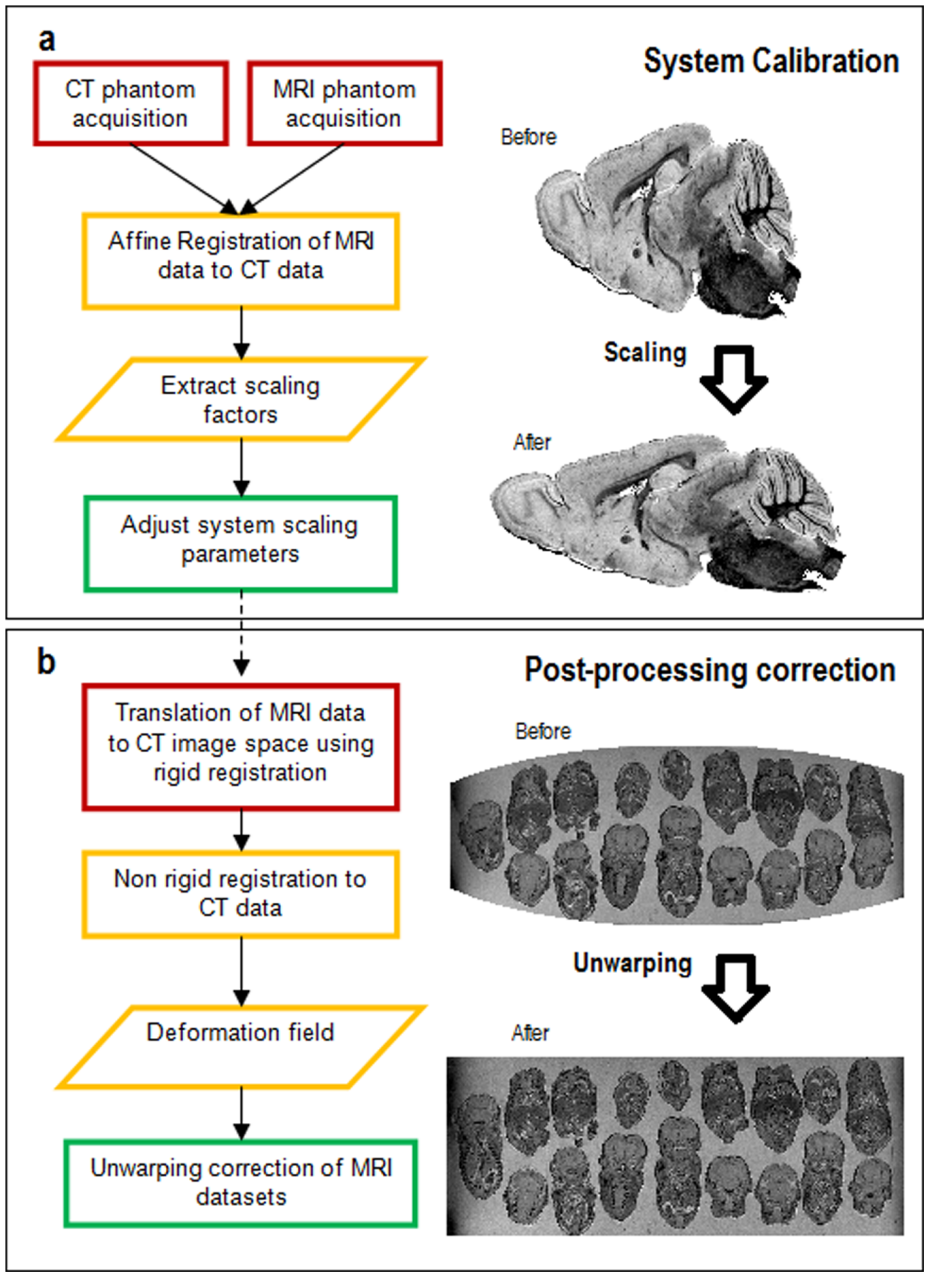
Gradient calibration protocol flowchart. Flowchart detailing the processes involved in the protocol and the order in which they should be implemented. The two major subdivisions of the technique are the system calibration (a) and the post-processing correction (b). Expected image deformations are illustrated using schematics.

The calibration protocol is shown schematically in Figure 1. Firstly, a 3D grid phantom is constructed using 3D printing, with dimensions set according to the size of the RF coil and/or scanner bore to be used. The phantom is then scanned using X-ray CT to provide a set of image data that is free from image distortion [6], [8], [9] (although for 3D printers with a high printing accuracy, the CAD plans for the phantom could be used as a fall back option if an X-ray CT system is not available). Next, scaling errors in the imaging gradients are corrected by performing a linear system calibration (Fig. 1a). Here, high-resolution, 3D gradient echo MR images of the grid phantom are acquired and are overlaid on the CT data using affine registration (i. e. scaling, translation, rotation and shearing). The registration parameters are then used to adjust the system gradient scaling values.

After system calibration of the scaling values, a second set of MR data is acquired, in order to perform a post-processing correction of gradient non-linearity. These data are translated into the CT imaging space using a rigid registration and then unwarped by non-rigid registration. This removes distortions caused by gradient non-linearity and any residual scaling errors that remain after the linear system calibration.

## Methods

### 3D Grid Phantom

A phantom containing a three dimensional grid structure was designed in SolidWorks (DSS Corp, Concord, MA) computer aided design (CAD) software and was 3D printed using a Formiga P100 plastic laser-sintering system (EOS Electro Optical Systems Ltd., Warwick, UK) at a layer thickness of 100 µm. It was manufactured using fine polyamide (PA-2200 nylon), a material with low water absorption properties (0.41% over an initial 24 hr period) and ability to withstand high mechanical and thermal load (EOS, PA 2200 Material Data Sheet). Nylon has a magnetic susceptibility that is close to that of water (<3 ppm) and so should not cause artefacts in either spin-echo or gradient-echo images [10].

**Figure 2:**
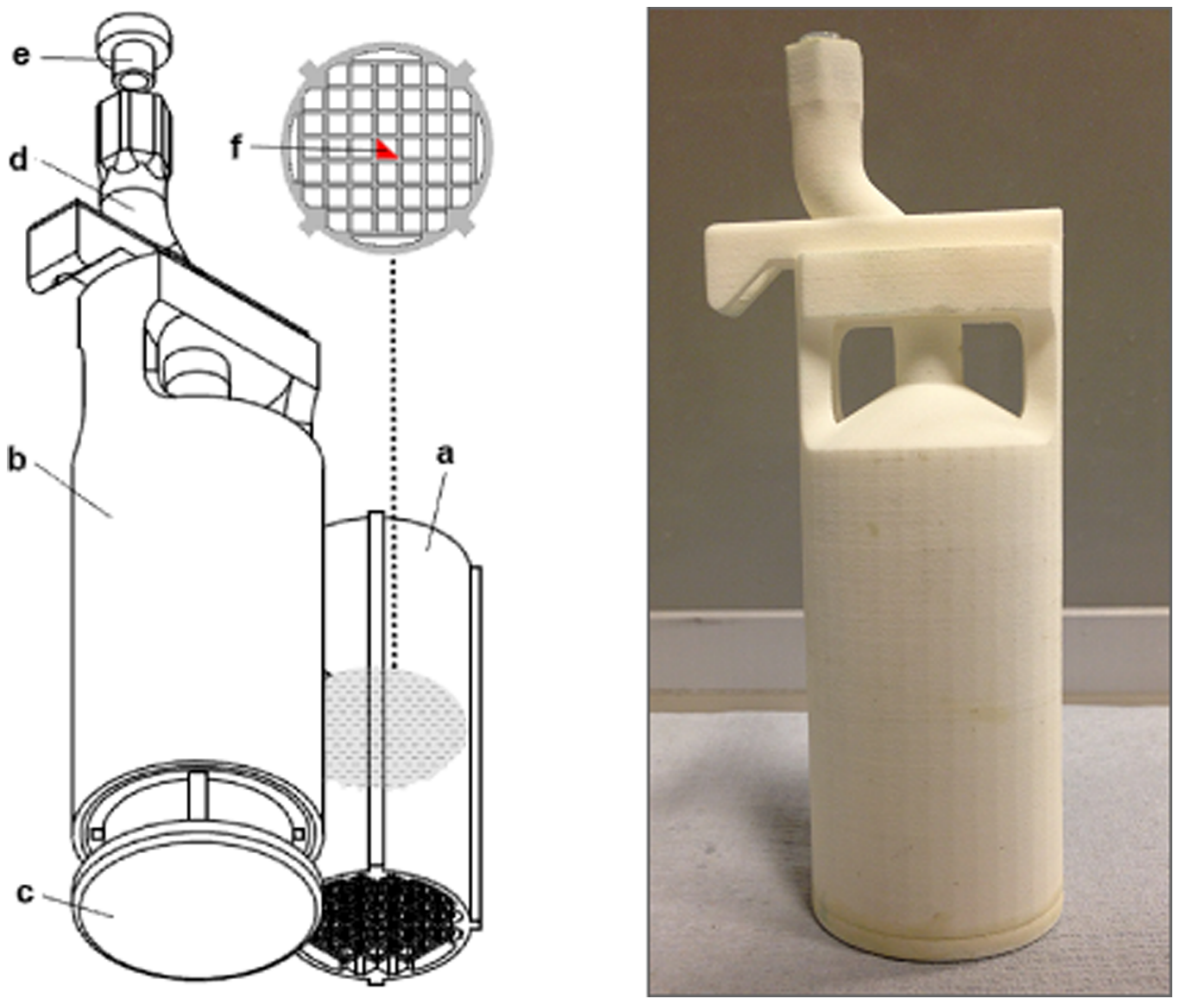
3D grid phantom design. CAD drawing of phantom sections and photograph of the assembled phantom, which was created using 3D printing. To assemble, the grid section (a) was inserted into the outer chamber (b) which was sealed by the chamber cap (c). It can then be filled through the s-bend (d) and sealed using a cap (e). An irregular prism (f) in the centre of the grid structure aids in the orientation of images.

For the current study, the dimensions of the phantom were 3D-printed to fit into a 35 mm birdcage coil that is routinely used for *in vivo* phenotyping of mice (Rapid Biomedical GmbH, Germany). The grid pattern occupied 75% of the diameter of the coil and its length (60 mm) encompassed its entire sensitive region (50 mm) and extended beyond the 40 mm DSV of linearity specified by the manufacturer. To enable removal of waste material generated during production, the phantom was formed in four pieces (Fig. 2). After cleaning, the grid section was inserted into the outer chamber, where it was sealed by attaching a chamber cap with epoxy resin. Also incorporated into the phantom design is a positioner with a thread for a nylon screw that allows consistent placement within the coil.

The walls of the three-dimensional grid pattern in the phantom were 0.5 mm thick to enable adequate sampling at the imaging resolution, and spaced 2.5 mm apart. An irregular prism (Fig. 2f) was included in the design of the phantom, at the centre of the grid, to aid orientation during post-processing. When inserted, the centre of the grid structure aligned with the isocentre of the magnet.

The phantom was filled with a solution of copper sulphate (1.25 g/L) and sodium chloride (5.3 g/L) in water (measured T_1_, 245 ms). The filling tube contained an s-bend shape to ensure air bubbles that accumulated at the top when stored in its vertical position were prevented from travelling into the main chamber during imaging.

### CT and MRI Imaging

**Figure 3:**
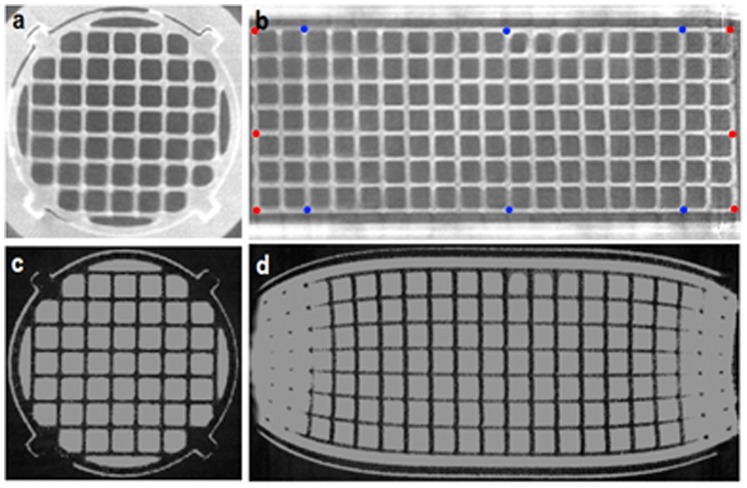
CT and MRI images of phantom. Axial (a) and coronal (b) slices from CT data and corresponding axial (c) and coronal (d) slices from 3D gradient echo MRI data. Landmarks for distance measurements are shown (b) for Z axis (red) and Y axis (blue) (landmarks in X axis are orthogonal to those in the Y axis at same Z coordinates).

CT images of the phantom (Fig. 3a, b) were acquired prior to filling with solution using a Bioscan nanoSPECT/CT system (Mediso, Budapest, Hungary). The Field of View (FOV) was selected to cover the whole grid section of the phantom and images were reconstructed using the vendor software to an isotropic resolution of 73 µm (system limit). To verify that the CT data were accurate, 10 manual measurements of the phantom were made using digital calipers and compared to CT data using NiftyView software (UCL, London, UK).

Prior to MRI acquisition, the phantom was filled and placed into the RF coil. An imaging gradient set with a 60 mm inner diameter was used in all experiments (SGRAD 115/60/HD/S, Agilent Technologies UK Ltd., Berkshire, UK), with a maximum gradient amplitude of 1 Tm^−1^. The gradient set is specified by the manufacturer to have a gradient sub-system rise time of 130 µs and a linearity of ≤5% within a 40 mm DSV.

All measurements were performed using a 9.4 T Agilent scanner, and images were acquired using a 3D gradient echo sequence, optimized for high resolution imaging of *ex vivo* murine brains [11]. The FOV was set to 26×30×30 mm^3^ in the central region of the phantom, within the 40 mm DSV of specified linearity. Voxel size was set to 40 µm, isotropic, and the readout gradient was 0.09 Tm^−1^, applied in the *Z* direction (parallel with the bore of the magnet). Other acquisition parameters included TR = 17 ms, TE = 4.54 ms, and a flip angle of 51°. Five averages were acquired, resulting in a total imaging time of 13 hours, 16 minutes. To investigate warping effects outside of the specified region of linearity and the possibility of reducing acquisition time for system calibration, data were also acquired at a reduced isotropic resolution of 100 µm over a larger FOV. The readout gradient was reduced to 0.04 Tm^−1^ and the FOV was 60×40×40 mm^3^, imaging the whole grid structure (Fig. 3c, d) in a reduced time of 3 hours, 46 minutes.

Shimming was performed manually at the start of each imaging session. The linewidth of the shim was monitored across imaging time points for consistency (40±5 Hz) using a pulse and collect sequence and the temperature variation during a scan was measured by attaching a probe to the outer surface of the phantom.

To investigate warping effects that may be specific to the gradient echo sequence implemented, 3D Fast Spin Echo images of the phantom were acquired over the larger FOV with an isotropic resolution of 100 µm. Other acquisition parameters included TR = 200 ms, Echo Train Length = 4, Echo Spacing = 6.86 ms, Averages = 5, and a read gradient strength of 0.11 Tm^−1^ applied in the Z direction (Acquisition time of 11 hours, 7 minutes). To maintain consistency in the comparison between sequences, gradient echo data was acquired at an isotropic resolution of 100 µm as detailed above with the read gradient strength increased to 0.11 Tm^−1^.

### System Calibration

NiftyReg software [12], [13], [14] was used for all image registration in this study. The affine and rigid registrations were carried out using the *reg_aladin* algorithm [12], [13] which employs a two step process. A block matching algorithm provides a set of corresponding points between a target and source image before the transformation is evaluated using normalized cross-correlation (NiftyReg webpages). To reduce processing time, initial alignment is assessed based on down-sampled low resolution images before final registration using full resolution images.

Prior to registration, the CT data was cropped reducing the FOV to include the grid section of the phantom only, providing initial alignment of the MRI and CT images. An affine transformation matrix was output by the *reg_aladin* algorithm, which was decomposed into its constituent transformation parameters. The gradient scaling values stored in the MRI console software were then adjusted through multiplication with the scaling parameters from the registration in the *X*, *Y* and *Z* directions.

Phantom grid structures were segmented within MRI data by intensity thresholding to remove high signal intensity voxels occupied by the filling solution, and was further refined by manual segmentation to include only the grid structure.

To evaluate the improvement in gradient scaling afforded by the affine registration, dice coefficients [15] of the segmented grid structures were compared, before and after adjustment. The dice coefficient, *s*, was defined as
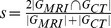
where 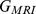 and 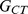 are the segmented grid structures of the MRI and the CT data. 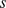 ranges from 0 (indicating no overlapping voxels) to 1 (indicating that all voxels overlap completely).

### Longitudinal Assessment of Calibration Accuracy

After the initial system calibration, MRI acquisitions were repeated at monthly intervals over a period of six months to measure stability over time. Additionally, CT images of the phantom were acquired at the same time points to account for structural deformations. To investigate global volume changes in the phantom, affine registrations of the CT data at each time point to the CT data acquired at the first time point were implemented, again using Niftyreg. Distortion in the structure was evaluated by taking distance measurements (using NiftyView) between grid section landmarks in the CT data (Fig. 3b), at three evenly spaced points along each axis. The measurements taken spanned the full diameter of the grid section in the *X*, and *Y* direction and the full length of the phantom in the *Z* direction. To test for a dependency between each of the measurements and longitudinal time point, a linear least squares regression analysis was performed to identify any significant correlation (*P* = 0.05, *ANOVA*).

### Post-Processing Correction

The MRI data (defined as the *source*) was transformed into the imaging space of the CT data (defined as the *target*) by rigid registration using the *reg_aladin* algorithm in NiftyReg. NiftySeg segmentation software was used to automatically generate a registration mask that encompassed the area within the outer casing of the phantom in the CT data. This prevented intensity changes outside of the grid section of the phantom affecting the registration. The *reg_f3d* algorithm [14] in NiftyReg was used to carry out a non-rigid registration to warp the MRI data, post rigid registration (*source*), to the CT data (*target*).

The *reg_f3d* algorithm [14] uses cubic B-splines to generate a deformation field. The local displacement of control points in an equally spaced lattice causes warping that modifies mapping between source and target images. The registration is assessed through Normalised Mutual Information as an indication of correspondence between images and warping is constrained through bending energy and elastic energy terms (NiftyReg webpages). Input parameters were optimized to generate a smooth deformation field. A spline grid spacing of 20 voxels was implemented and the weight of the linear elastic energy penalty term was increased to [0.01 0.01].

To investigate the reproducibility of the results, the phantom was imaged four times, back-to-back, and displacement fields were generated for each scan. The first acquisition was taken as a reference and the absolute displacement differences at each point were calculated in the *X*, *Y*, and *Z* orthogonal directions (*Z* is aligned with the axis of the scanner bore), for each subsequent acquisition.

## Results

### Phantom Stability Measurements

The structural stability of the phantom was assessed over a six month period from CT data acquired every month. The maximum change in scaling identified on any axis was 0.28%. Phantom length measurements showed no significant correlation between variables, which suggests that there were no gradual changes in the phantom structure over time. The comparison of manual measurements in the CT imaging data to those made using digital callipers at a single time point showed a mean percentage difference of 0.27±0.17%, indicating a close agreement. The temperature of the phantom recorded during an MRI acquisition ranged between 19.9°C and 20.2°C (melting point of PA 2200 is 184°C).

### System Calibration

**Figure 4:**
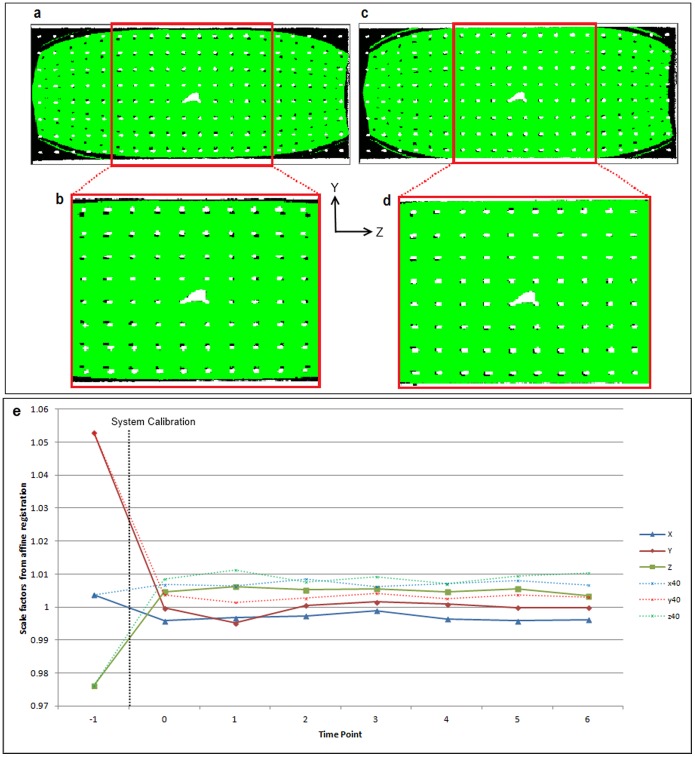
Gradient scaling values before and after system calibration. Sagittal slices of CT phantom images (white) overlaid on MRI images (filler inside phantom shown in green, phantom structure shown in black, 26×60 mm FOV ) showing alignment before (a, b) and after (c, d) system calibration. The errors in the scaling factors (e) prior to calibration (time point −1) are reduced after system adjustment (TP 0) and the factors calculated using 100 µm data (X, Y, Z) are in good agreement with the 40 µm data (x40, y40, z40).

Prior to the start of the study, the imaging gradients of the 9.4 T scanner had been calibrated using the scanner manufacturer’s standard protocol described in the introduction. Details of this protocol are published in a user manual supplied with their structural phantom. By comparing the CT phantom images with 100 µm-resolution MRI data (acquired with the manufacturer’s calibration) after rigid registration, we observed marked discrepancies between CT and MRI images (white and black structures, respectively, in Figs. 4a and b). Relative to CT, MRI images were globally compressed in the *Y* direction and expanded in the *Z* direction. This can be seen most clearly in a central section of the phantom, within the 40 mm DSV specified by the manufacturer, where gradient linearity is optimal (Fig. 4b). Figure 4e demonstrates the magnitude of these global changes, which varied with direction (scaling*: X* = 100.4%, *Y* = 105.3%, *Z* = 97.6%). This experiment was defined as scaling time point −1.

Outside the 40 mm DSV, it was clear that discrepancies between MRI and CT data markedly increased due to gradient non-linearity. To reduce the impact of this distortion when comparing image distortion before and after calibration, dice coefficients were only calculated in a region of interest corresponding to the central section of the phantom, within the 40 mm DSV (Fig. 4b). Correction of image distortion due to gradient non-linearity is the purpose of the *post-processing correction* evaluated later in the study.

The system was then calibrated using our proposed protocol (scaling time point 0), a new set of MRI data was acquired, and rigid registration to the CT data was repeated. The accordance between these data and CT prior to and following calibration (Fig. 4) was assessed by comparing dice coefficients, *s*: in the central region of interest, *s* was 0.73 before calibration and 0.84 afterwards (0.57 and 0.67, respectively, over the whole FOV). Affine registration of the MRI data acquired after the system calibration to the CT data produced scaling factors of 99.6%, 99.9%, 100.5% in the *X*, *Y*, and *Z* axes (Fig. 4e), which is a marked improvement over the pre-calibration values. The mean scaling factor error (relative to unity) across all axes after correction was reduced from 2.7% to 0.3%.

Using the same approach as described above, the mean and standard deviation of the scaling factors measured every month, for a total of six months, were 99.7±0.1%, 100±0.2%, and 100.1±0.1% in the *X, Y* and *Z* directions.

The scaling factors calculated by registering the MRI data acquired at an isotropic resolution of 40 µm to the CT data followed a very similar pattern to the 100 µm MRI data (Fig. 4e). The mean percentage difference between the 40 µm and the 100 µm scaling factors taken across all time points was 0.52±0.23%.

### Post-processing Correction

**Figure 5:**
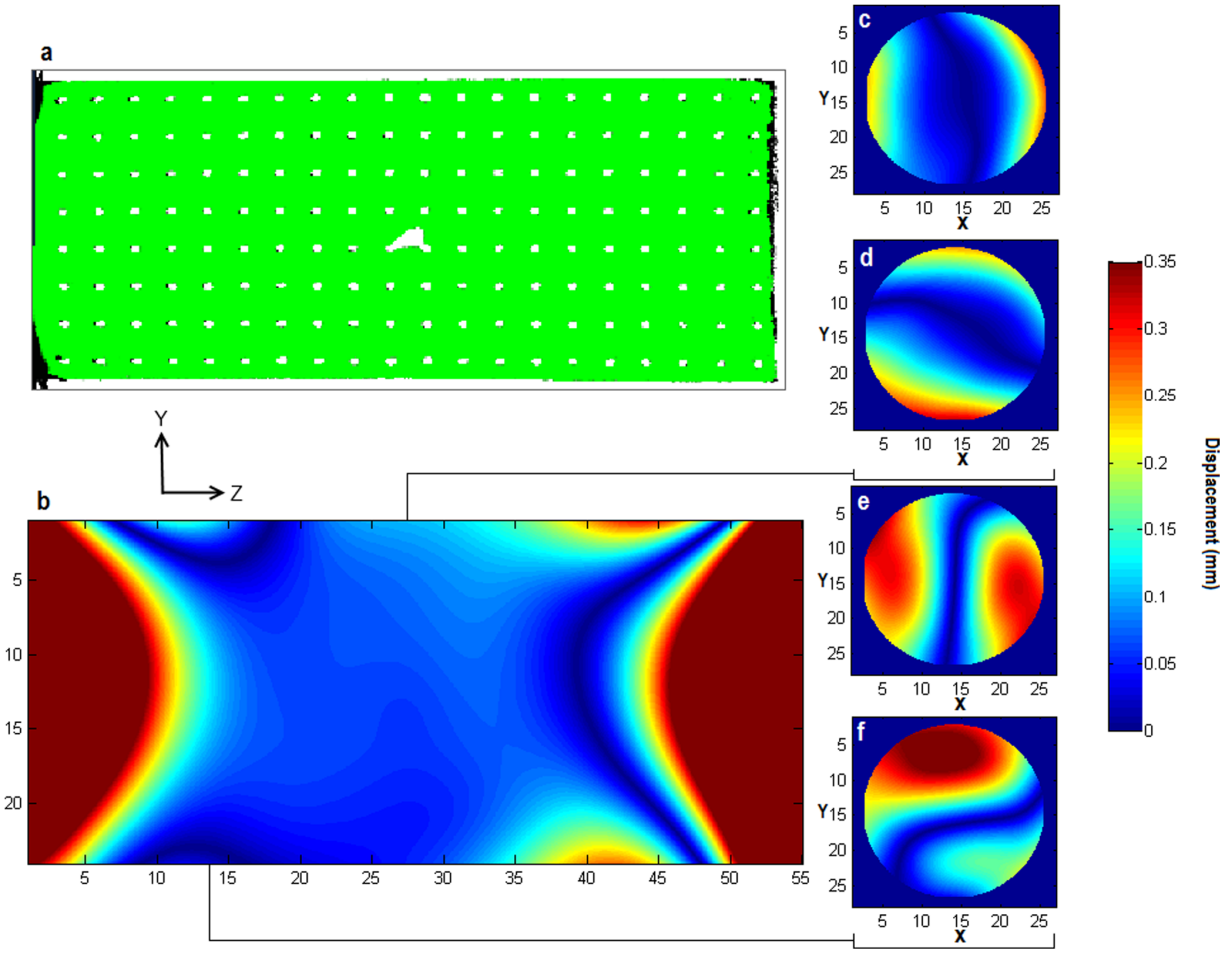
Displacement fields generated from post-processing correction. Projection along the X axis from CT phantom data (white) overlaid on MRI images (filler inside phantom shown in green, phantom structure shown in black) show good alignment after post-processing correction (a). Displacement fields generated from the non-rigid registration show displacements increase along the Z axis (b) as distance from the centre increases. This is also observed in the X (c) and Y (d) axes for central slices. Slices taken further from the centre show the displacements in the X (e) and Y (f) directions increase more rapidly with distance from the slice centre.

Alignment of MRI and CT data was further improved after non-rigid registration (Fig. 5a), especially in regions further from the magnet isocentre. The dice coefficient calculated from the whole grid structure in the phantom increased from 0.62 before post-processing correction, to 0.88.

A displacement field, generated from the deformation field output by the non-rigid registration, shows the magnitude of voxel displacements applied to the MRI data to unwarp and register it to the CT data (Fig. 5b–f). The displacements along the *Z* axis (Fig. 5b) were less than 0.1 mm at a distance of less than ±10 mm from the isocentre. At a distance of ±20 mm, and greater, the displacements increase rapidly to more than 0.35 mm. The displacements along the *X* and *Y* axes show small displacements of less than 0.1 mm in central slices (Fig. 5c, d) at distances of less than ±5 mm from the centre. For slices located further from the isocentre (in the *Z* direction) (Fig. 5e, f), the magnitude of displacements markedly increases. Outside the central region of relatively low displacement, there is a rapid increase to values larger than 0.25 mm in the *X* and *Y* direction within the walls of the grid section of the phantom.

Within the central 40 mm DSV, where linearity is specified by the manufacturer, the maximum displacement was 0.72 mm, 0.87 mm and 0.6 mm in the *X*, *Y*, and *Z* axes, corresponding to a linearity of 3.6%, 4.35%, and 3% respectively. The maximum displacements in the phantom outside of this volume were 3.51 mm, 3.81 mm, and 2 mm in the *X, Y* and *Z* axes. The repeated generation of displacement fields to test reproducibility revealed the mean discrepancy between calculated displacements to be 11.8±10.2 µm across all axes for the whole FOV.

### MRI Sequence Comparison

Scaling factors calculated using a gradient echo sequence and a fast spin echo sequence showed a percentage difference of 0.06%, 0.02%, and 0.03% in the *X*, *Y*, and *Z* axes respectively. The mean displacement difference taken across the whole grid structure was 19±30 µm.

## Discussion

The precision and stability of a pre-clinical MRI system are of paramount importance when performing quantitative, comparative and longitudinal measurements in imaging subjects over time. We have developed a gradient calibration protocol, specific to pre-clinical imaging systems, that can quantify and correct for these errors. Moreover, we have shown that, for the accuracy required for the detection of microscopic changes in tissue structure and size, significant measurement errors can be introduced through imperfect gradients, particularly when relying on calibration protocols with low acceptance limits. Our protocol is based on a 3D-printed geometric phantom, featuring a three-dimensional grid structure. The plans for the phantom have been published online and can easily be adapted for individual RF coils and scanner bore sizes. The phantom design contains novel features such as an s-bend in the filling pipe to prevent air bubbles in the main cavity, and the ability to attach as a single piece directly to the RF coil to improve consistency in phantom positioning between measurements.

Monitoring of relative gradient scaling factors using MRI data has been reported previously [7]. In this study we used high-resolution CT and MRI imaging data to validate the structural stability of the phantom over time and provided a simple method for the absolute scaling of the system gradients. The ability to reduce errors in scaling values will improve accuracy of measurements acquired on the system and invites the possibility of reuse of control group data reducing animal numbers. We have also introduced a post-processing technique for the correction of image distortions caused by gradient non-linearity.

Relative to the scanner manufacturer’s standard calibration, our system calibration reduced the mean gradient scaling factor error from 2.7% to 0.3%. Errors of the magnitude found prior to our calibration have the potential to be a significant confounder to detection of structural volume changes in the mouse brain, which can be less than 2% [1].

The calibration requires the acquisition of three-dimensional gradient echo data, and we compared the accuracy of acquiring at 40 and (more rapid) 100 µm isotropic resolution. There was close agreement between the scaling parameters calculated from gradient echo data acquired at two different resolutions and fast spin echo data, indicating that accurate system calibration can be performed using the gradient echo protocol with reduced scan time for inclusion in a routine QA protocol. Application of such a protocol on pre-clinical systems is clearly important, particularly given the magnitude of gradient errors that resulted from the manufacturer’s standard calibration. A 5.3% scaling error was found in the *Y* direction prior to calibration, which, for example, would result in a 9.8% error in apparent diffusion coefficient (ADC) estimates, calculated from DWI data (due to the inverse square relationship between b-value and gradient magnitude). Moreover, the scaling values, once corrected with our protocol, were stable over the six month period, indicating that it may be satisfactory to carry out as few as two system calibrations per annum.

We found that displacements near to the isocentre of the magnet, following calibration, can be less than 0.1 mm. The linearity of the gradient set used was specified by the manufacturer as ≤5% within a central 40 mm DSV region. The measured linearity was found to be within these limits in each axis. However, this tolerance corresponds to a maximum spatial deviation of 1 mm which may not be satisfactory for phenotyping applications and a correction may be required. Outside of this region, image distortion increases rapidly, with displacements of 0.3 mm and larger. With the application of non-rigid registration during post-processing, the dice coefficient improved by 26%. The strong correspondence between the CT data and the corrected MRI indicates that non-rigid registration approach is a robust solution to unwarping data in regions of large distortion.

The use of the generated deformation field may reduce distortions significantly, especially when imaging samples such as multiple embryos or anatomy that is positioned at a distance from the magnet isocentre. Therefore, the post-processing correction for gradient non-linearity can increase the effective FOV over which biological samples can be accurately imaged, markedly increasing the efficiency of high resolution scans that are often acquired overnight. Assuming satisfactory stability of the gradients, the deformation field from a single time point could be used to correct multiple datasets collected over a six month period for animal phenotyping studies [16].

In this study, MRI acquisition parameters were based on an *ex vivo* murine structural neuroimaging sequence with the aim of correcting this data using the deformation field. The use of a 3D gradient echo sequence to characterise gradient field distortion is in line with previous clinical studies [2], [6], [8], [17], [18] and the agreement of scaling factors calculated from data acquired at two resolutions and using a fast spin echo sequence indicate robustness of the methodology to changes in the read gradient magnitude and MRI sequence. The mean difference in displacements between the gradient echo and fast spin echo data is within a single standard deviation of the reproducibility data mean difference, suggesting that the warping, caused by non-linearity of the gradients, is dominating any sequence specific effects in the scenario investigated.

The deformation field could also be used to unwarp *in vivo* data sets collected with the same imaging protocol although it should be noted that some variability in the accuracy of spatial displacement may be introduced by sample-dependent B0 perturbations. These should be minimal in structural imaging and if necessary can be corrected for through the use of existing techniques [19]. Minor deviations from the protocol described here may be necessary such as adjustments to the phantom dimensions to fit specific hardware configurations and an alteration of the composition of the phantom filler solution to optimize SNR for the particular pulse sequence used.

In this work we present a complete protocol consisting of a system calibration of MRI gradients and a post-processing correction for non-linearities away from the magnet isocentre. The phantom design is open-source and can be adjusted as necessary for the specific imaging protocol, RF coil and scanner dimensions used. The NiftyReg software used for the system calibration and the post-processing correction is also freely available to download and has been used to perform absolute scaling of gradients and an image correction of distortions caused by gradient non-linearity. This simple step-by-step process can be integrated with or form the basis of a QA protocol that could be implemented during installation and as part of routine maintenance on any pre-clinical MRI system.
